# Neuropsychiatric disorders in chronic hepatitis C patients after receiving interferon or direct-acting antivirals: a nationwide cohort study

**DOI:** 10.3389/fphar.2023.1191843

**Published:** 2023-07-19

**Authors:** Yu Fang, Chung-Yu Chen, Hsien-Chung Yu, Pei-Chin Lin

**Affiliations:** ^1^ Master Program in Clinical Pharmacy, School of Pharmacy, Kaohsiung Medical University, Kaohsiung, Taiwan; ^2^ Department of Pharmacy, Pingtung Veterans General Hospital, Pingtung, Taiwan; ^3^ Department of Pharmacy, Kaohsiung Medical University Hospital, Kaohsiung, Taiwan; ^4^ Department of Medical Research, Kaohsiung Medical University Hospital, Kaohsiung, Taiwan; ^5^ Health Management Center, Kaohsiung Veterans General Hospital, Kaohsiung, Taiwan; ^6^ Division of Gastroenterology and Hepatology, Department of Internal Medicine, Kaohsiung Veterans General Hospital, Kaohsiung, Taiwan; ^7^ Faculty of Medicine, School of Medicine, National Yang-Ming University, Taipei, Taiwan; ^8^ Department of Business Management, Institute of Health Care Management, National Sun Yat-sen University, Kaohsiung, Taiwan; ^9^ Department of Nursing, Meiho Institute of Technology, Ping-Tung, Taiwan; ^10^ Department of Medical Education and Research, Kaohsiung Veterans General Hospital, Kaohsiung, Taiwan; ^11^ School of Pharmacy, Kaohsiung Medical University, Kaohsiung, Taiwan

**Keywords:** hepatitis C virus, direct-acting antivirals, neuropsychological disorders, anxiety, depression

## Abstract

**Background:** Data on the neuropsychological outcomes after receiving direct-acting antivirals (DAAs) among chronic hepatitis C (CHC) patients have not been well-documented.

**Aim:** This study aimed to evaluate the difference in incidence of neuropsychological disorders (NPDs) after treatment completion between CHC patients receiving interferon (IFN) therapy and DAA therapy.

**Methods:** A nationwide retrospective cohort study was performed using Taiwan’s National Health Insurance Research Database (NHIRD) between 2010 and 2018. CHC patients without pre-existing mental disorders were included and divided into the treatment (Tx)-naïve DAA group, retreatment (re-Tx) DAA group, and Tx-naïve IFN group based on their HCV therapy. Propensity score matching was used to balance baseline differences between groups. The primary outcome was the incidence of NPDs during 6 months after completion of therapy.

**Results:** After one-to-one matching, there were 6,461 pairs of patients selected from the Tx-naïve DAA group and Tx-naïve IFN group and 3,792 pairs from the re-Tx DAA group and Tx-naïve IFN group. A lower incidence of NPDs was observed in the Tx-naïve DAA group than in the Tx-naïve IFN group (HR = 0.72, 95% CI = 0.55–0.94, and *p* = 0.017). The risk of NPDs did not differ between the re-Tx DAA group and the Tx-naïve IFN group (HR = 0.74, 95% CI: 0.52–1.05, and *p* = 0.092).

**Conclusion:** DAA therapy was associated with lower risk of NPDs when compared with IFN therapy among Tx-naïve CHC patients in a 6-month period after treatment completion, especially among the patients less than 65 years, male gender, and cirrhosis.

## 1 Introduction

Hepatitis C virus (HCV) infection is a major global health issue, which leads to hepatic complications and several extrahepatic manifestations, including neurologic and neuropsychiatric diseases (Cacoub and Saadoun, 2021). The estimated global HCV prevalence in 2015 was 1%, which corresponded to 71.1 million infected individuals worldwide (Polaris Observatory HCV Collaborators, 2017). In Taiwan, the prevalence of HCV infection was approximately 2.1% in 2015, which was higher than the global average (Chen et al, 2007; Polaris Observatory HCV Collaborators, 2017). Symptoms of fatigue, depression, and cognitive impairment are commonly reported in chronic hepatitis C (CHC) patients (Poynard et al., 2002; Golden et al., 2005; McAndrews et al., 2005). In a population-based study, a 1.36-fold higher risk for dementia was observed in HCV-infected patients (Chiu et al., 2014).

Such neuropsychological disorders (NPDs) had been hypothesized to be related to a direct HCV infection of the central nervous system (CNS) or neurological damage secondary to HCV-related systemic inflammation (Laskus et al., 2005; Senzolo et al., 2011). Evidence for HCV infection of the CNS was supported with detection of active HCV replication in cerebrospinal fluid, which leads to neuroinvasion and neuroinflammation (Bolay et al., 1996; Laskus et al., 2002; Neal, 2014; Maximova and Pletnev, 2018). However, the exact pathophysiological mechanisms remain poorly understood, and there was no clear correlation between HCV viral load and risk of NPDs (Martindale et al., 2017). The adverse effect associated with interferon therapy might also be a contributing factor (Zdilar et al., 2000; Dieperink et al., 2003; Horikawa et al., 2003; Kraus et al., 2003). Since the evidence suggested a pathogenic role for the HCV virus itself, eradication of HCV infection with antiviral treatment may be associated with an improvement in neuropsychological symptoms. Before the approval of the direct-acting antiviral agents (DAAs) for HCV treatment in 2014, all recommended HCV regimens included interferon (IFN) and ribavirin (RBV) (Rockstroh, 2015).

A prospective one-arm study was conducted to evaluate the impact of sustained virologic response (SVR) on cognitive function and mood disorders in CHC patients treated with IFN, of which the results suggested that eradication of HCV infection with interferon treatment improved cognitive performance, whereas no improvement in depressive symptoms was observed (Barbosa et al., 2017). In a nationwide cohort study evaluating the impact of successful IFN therapy on the incidence of major psychoses in Taiwanese CHC patients, the 10-year cumulative incidence of schizophrenia was significantly lower in the SVR group than in the non-SVR group (*p* = 0.036) (Tsai et al., 2020). However, the incidence of affective psychosis was not statistically different between the SVR and non-SVR groups (*p* = 0.667).

Since the introduction of DAAs, a breakthrough in HCV treatment was achieved with high rates of sustained virologic response and improved tolerability (Götte and Feld, 2016). The neuropsychological outcome after DAA therapy has also been evaluated. Most of recent observational studies using scale measurement showed CHC patients treated with DAAs experienced trends for improved outcome (Gallach et al., 2018; Sundberg et al., 2018; Evon et al., 2019; Goñi Esarte et al., 2019; Kesen et al., 2019; Fabrazzo et al., 2020; Vaghi et al., 2020). In a multicenter observational study of 1601 CHC patients treated with DAAs between 2016 and 2017, post-treatment scores in domains of depression, anxiety, and cognitive concern showed significant improvements from the baseline scores among the SVR cohort (Evon et al., 2019).

In summary, related research suggested eradication of HCV infection with either IFN or DAA treatment was associated with improved neuropsychological outcome in CHC patients. However, the neuropsychological outcome was measured with a self-control scale survey in previous studies, and the majority of studies that included the HCV cohort treated with DAAs were of small sample size, and the results need to be validated in a larger sample. There is lack of evidence to support whether DAAs reduce the risk of developing new cases of neuropsychological disorders among CHC patients. Therefore, the present study was performed to investigate whether DAA therapy reduces the risk of neuropsychological disorders in a nationwide sample of CHC patients.

## 2 Methods

### 2.1 Data source

This retrospective cohort study was conducted using Taiwan’s National Health Insurance Research Database (NHIRD), which contains comprehensive healthcare information of insured individuals, including basic demographic data, dates of clinical visits, diagnostic codes, prescription details, operations, and examinations. The NHI program was launched in 1995 and currently provides reimbursements for healthcare costs for more than 99% of the current population in Taiwan (23 million) (National Health Insurance Administration, 2018).

### 2.2 Study population and design

**FIGURE 1:**
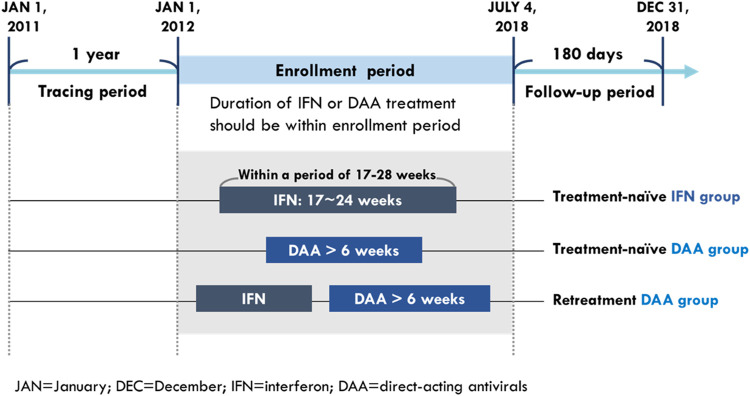
Eligibility for study enrollment.

The study population comprised treatment (Tx)-naïve CHC patients who received DAA or IFN treatment and retreatment (re-Tx) patients who received DAA and had a prior IFN failure. The CHC patients were defined as those who had one or more diagnoses of HCV infection (according to the ICD-9-CM codes: 070.2, 070.3, and V02.61 and ICD-10-CM codes: B17.1, B18.2, B19.2, and Z22.52) in an outpatient or inpatient setting and had records of prescriptions for HCV medication including either IFN (according to the ATC codes: L03AB04, L03AB05, L03AB09, L03AB10, and L03AB11) or DAAs (according to the order codes: HCVDAA0001–HCVDAA0016) during the study period from 2011 to 2018. To ensure the observation period was long enough for identifying baseline characteristics and study outcomes, the time from initiation to last exposure of their HCV medications should be within the enrollment period, which was between 1 January 2012 and 4 July 2018 (Figure 1).

The CHC patients who received DAAs were categorized on the basis of IFN exposure before initiation of DAA treatment: those without histories of IFN prescription were included in the Tx-naïve DAA group and those with histories of IFN prescription were included in the re-Tx DAA group. CHC patients who received DAAs and had no histories of IFN prescription were included in the Tx-naïve DAA group. Under the policy of Taiwan’s NHI system, the course of DAA treatment is reimbursed for up to 6 weeks when CHC patients fail to achieve two-log reduction of viral load at week 4 during DAA treatment. Therefore, patients whose cumulative medication duration of DAA treatment was more than 6 weeks were included in the Tx-naïve DAA group and re-Tx DAA group.

Among patients who only received IFN therapy, patients treated with a single course of treatment were included in the IFN group. To compare the IFN group more with the DAA groups, potentially well-tolerant IFN users with rapid viral response (RVR) were selected from Tx-naïve IFN users for comparison group with the DAA groups. According to the reimbursement policy of NHI, patients were eligible for a second IFN treatment when relapses occurred after 24 weeks following the completion of a successful IFN treatment. The reimbursement duration of IFN treatment based on the policy of the NHI system was based on virological response; IFN treatment is reimbursed for up to 24 weeks for patients showing RVR and up to 16 weeks for those who failed to achieve rapid viral response (RVR). Therefore, patients were identified as Tx-naïve IFN users if the gap between their IFN prescription durations was not 24 weeks or more. Among Tx-naïve IFN users, patients with cumulative supply of their IFN prescriptions between 17 and 24 weeks in a duration of 17–28 2.2weeks were considered to be well-tolerated patients with RVR and included in the IFN group.

Patients were excluded if they (1) were aged less than 20 years on the index date; (2) had missing information on age and gender; (3) received IFN after initiation of DAA therapy; (4) with pre-existing mental disorders; (5) died or had study outcomes during HCV treatment. The index date was defined as the date of initial prescription of DAA for patients in both the Tx-naïve and re-Tx DAA groups, and the date of initial prescription of IFN was designated as the index date for those in the IFN group. Patients with pre-existing mental disorders (according to the ICD-9-CM codes: 290–319 and ICD-10-CM codes: F00–F99) were identified based on one inpatient or two outpatient diagnoses within 1 year before the index date. Baseline characteristics were measured during the year before the index date. Comorbidities were identified as one inpatient or two outpatient diagnoses (Supplementary Table SA1). Concomitant medications were identified as the total days of supply from their prescriptions, which was more than 28 days within 6 months (180 days) before the index date.

### 2.3 Outcome measurements

The primary outcome was the incidence of NPDs, which was defined as a composite of one outpatient or inpatient diagnosis of mood and anxiety disorders, psychotic disorders, and cognitive disorders. The secondary outcome included mood and anxiety disorders, and other NPDs. The mood and anxiety disorders included depressive disorders, mania, bipolar disorders, and anxiety disorders. The psychotic disorders of interest included schizophrenia, schizotypal, and delusional disorders. The cognitive disorders of interest include Alzheimer’s disease, dementia, and delirium. Diagnosis codes for study outcomes are described in the appendix (Supplementary Table SA3). The study outcomes were identified during the follow-up time of 6 months after the end of the HCV treatment. Patients who did not develop NPDs were censored at death or at the end of follow-up, whichever happened first.

### 2.4 Statistical analysis

We calculated the frequency and percentage for the categorical variables. Continuous variables were expressed as mean and standard deviation (SD) and median and interquartile range (IQR). Differences between two groups were assessed using standardized mean differences (SMD), which indicate a negligible difference when the value is less or equal to 0.1. The patient adherence in the IFN group was estimated according to the sum of week supply of IFN during the treatment period divided by the total number of weeks of the treatment period, which is defined as good adherence if the valued is more than or equal to 80% (European Association for Study of Liver, 2014). In both the Tx-naïve and re-Tx DAA groups, good adherence was determined by whether individuals had completed the planned duration of DAA treatment. The planned duration of DAA treatment could be identified according to the specific order codes for DAA regimens, which corresponded to respective reimbursed durations.

Propensity score matching was used to balance the baseline difference between treatment groups and address the potential for treatment selection bias. Each patient in the Tx-naïve DAA group and re-Tx DAA group was assigned one matched control in the Tx-naïve IFN group based on the logit of the propensity score using calipers of width equal to 0.2 SD of the logit of the propensity score. The propensity score was generated based on the probability that an individual would receive DAA treatment and computed by a multivariate logistic regression model adjusting for baseline covariates, including age group, gender, cirrhosis, liver cancer, and other variables which showed significance (*p* < 0.05) in univariate Cox regression analysis for risk of NPDs among the crude cohort.

The crude incidence rate of neuropsychological disorders for each group was calculated according to the number of events in the follow-up period divided by the total follow-up (per 1,000 person-months). The cumulative incidence curve was estimated using the Kaplan–Meier approach, and Gray’s test was used to investigate group differences. Because death is a competing risk event for neuropsychological disorders, Cox proportional hazards models with competing risks (cause-specific hazard model) were performed to estimate the hazard ratio (HR) and 95% confidence intervals (CI) of NPDs between treatment groups. According to epidemiological studies (Wittchen et al., 2011; Niu et al., 2017; Hasin et al., 2018), female gender and younger age have been found to be factors associated with an increased risk of depression and anxiety. Additionally, increasing age has been the strongest known risk factor for dementia. Therefore, multivariate analysis was performed to adjust gender, age, and other selected baseline variables. Variable selection strategies were performed to reduce the interaction between covariates. First, variables which showed significant differences (*p* < 0.05) in the univariate analysis were selected as candidate variables. Second, a further selection with stepwise regression included covariates of gender, age, and candidate variables. The probability for stepwise regression in the removal was 0.05. Finally, the selected variables in stepwise regression as well as age and gender were included as covariates in the multivariate analysis. Subgroup analysis was performed to further evaluate the difference in the risk of NPDs between treatment groups after stratifying by age, gender, and status of liver cirrhosis. All the aforementioned analyses were conducted using SAS statistical software (version 9.2).

### 2.5 Ethical considerations

This study was conducted using NHIRD and previously approved by the the Institutional Review Board of Kaohsiung Medical University Hospital (IRB number KMUHIRB-E(I)-20210137). Data in NHIRD were de-identified by scrambling the identification codes of patients and care providers before being released to each researcher.

## 3 Results

### 3.1 Baseline characteristics

**FIGURE 2:**
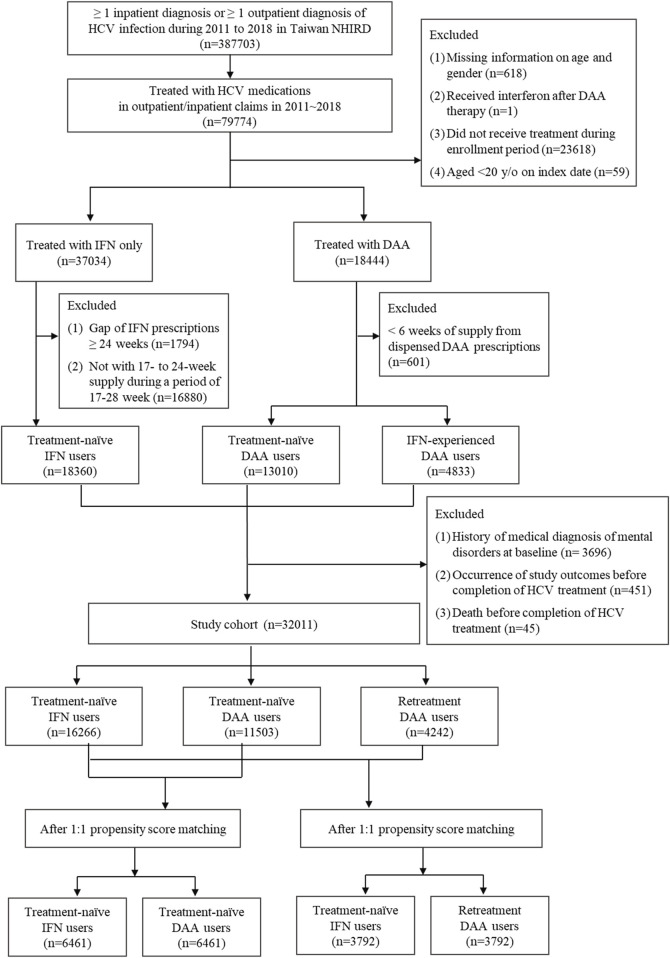
Study population attrition flow chart.

A total of 79774 CHC patients received IFN or DAA therapy during the period of 2012–2018. Based on the inclusion and exclusion criteria, 32840 patients were included in this study, including 16657 patients in the IFN group, 11809 patients in the Tx-naïve DAA group, and 4,374 patients in the re-Tx DAA group. After employment of one-to-one matching, there were 6,461 matched pairs generated in the study population comprising the Tx-naïve DAA group and IFN group, and 3,792 matched pairs generated in those comprising the re-Tx DAA group and IFN group. The study flowchart is depicted in Figure 2.

The baseline characteristics of Tx-naïve and re-Tx DAA groups and their matched controls in the IFN group are presented in Table 1. The distribution of gender, age group, cirrhosis, and liver cancer was comparable between matched patients in the Tx-naïve DAA group and IFN group, as well as those in the re-Tx DAA group and IFN group. The mean age of patients was 61.9 (±9.6) years for the Tx-naïve DAA group and 60.9 (±9.3) 3years for the IFN group (SMD = 0.092). The prevalence of medication use of histamine 2 receptor antagonists and beta-blockers was higher in the Tx-naïve DAA group. There were more patients in the Tx-naïve DAA group than in the IFN group with medication use of histamine 2 receptor antagonists (21.4% vs. 16.4% and SMD = 0.127) and beta-blockers (22% vs. 17.6% and SMD = 0.109). In the matched cohort of the re-Tx DAA group and IFN group, the mean age of patients in the re-Tx DAA group was 62.8 (±9.3) years, and the mean age of patients in the IFN group was also 62 (±9.3) years (SMD = 0.084). There were also no significant differences in the distribution of comorbidities and co-medications between matched patients in the re-Tx DAA group and IFN group. More than 96% patients in both the Tx-naïve and re-Tx DAA groups had completed the planned duration of DAA treatment. Nearly all controls (99.8%) in the IFN group for the Tx-naïve and re-Tx DAA groups had an adherence rate of more than 80%.

**TABLE 1:** Baseline characteristics of study cohorts after PSM.

	Cohort I		SMD	Cohort II		SMD
	IFN (n = 6,461)	DAA (n = 6,461)		IFN (n = 3,792)	reTx—DAA (n = 3,792)	
	n (%)	n (%)		n (%)	n (%)	
**Male**	3,242 (50.2)	3,242 (50.2)	0.092	1761 (46.4)	1761 (46.4)	<0.001
**Age**, mean ± SD	60.87 ± 9.3	61.75 ± 9.6	<0.001	62.02 (9.3)	62.79 (9.3)	0.084
Age <40	119 (1.8)	119 (1.8)	<0.001	58 (1.5)	58 (1.5)	<0.001
40≤ age<50	571 (8.8)	571 (8.8)	<0.001	265 (7)	265 (7)	<0.001
50≤ age<60	1804 (27.9)	1804 (27.9)	<0.001	933 (24.6)	933 (24.6)	<0.001
60≤ age<70	2767 (42.8)	2767 (42.8)	<0.001	1,675 (44.2)	1,675 (44.2)	<0.001
70≤ age<80	1,151 (17.8)	1,151 (17.8)	<0.001	817 (21.6)	817 (21.6)	<0.001
Age ≥80	49 (0.8)	49 (0.8)	<0.001	44 (1.2)	44 (1.2)	<0.001
**Comorbidity**						
Hypertension	1,032 (16)	1,148 (17.8)	0.048	681 (18)	673 (17.8)	0.006
Hyperlipidemia	735 (11.4)	716 (11.1)	0.009	440 (11.6)	417 (11)	0.019
Diabetes mellitus	658 (10.2)	574 (8.9)	0.044	423 (11.2)	437 (11.5)	0.012
Chronic kidney disease	128 (2)	188 (2.9)	0.06	85 (2.2)	89 (2.4)	0.007
Ischemic heart disease	194 (3)	219 (3.4)	0.022	114 (3)	116 (3.1)	0.003
Cerebrovascular disease	84 (1.3)	72 (1.1)	0.017	51 (1.3)	49 (1.3)	0.005
Autoimmune disorders	247 (3.8)	202 (3.1)	0.038	145 (3.8)	113 (3)	0.047
Cirrhosis	393 (6.1)	393 (6.1)	<0.001	288 (7.6)	288 (7.6)	<0.001
Liver cancer	193 (3)	193 (3)	<0.001	152 (4)	152 (4)	<0.001
HBV coinfection	194 (3)	174 (2.7)	0.019	88 (2.3)	110 (2.9)	0.036
**CCI**, mean ± SD	2.35 ± 1.4	2.39 ± 1.4	0.025	2.54 (1.4)	2.58 (1.5)	0.029
0	443 (6.9)	443 (6.9)	<0.001	206 (5.4)	206 (5.4)	<0.001
1	1,234 (19.1)	1,234 (19.1)	<0.001	618 (16.3)	618 (16.3)	<0.001
2	2080 (32.2)	2080 (32.2)	<0.001	1,147 (30.3)	1,147 (30.3)	<0.001
3	1,616 (25)	1,616 (25)	<0.001	998 (26.3)	998 (26.3)	<0.001
≥4	1,088 (16.8)	1,088 (16.8)	<0.001	823 (21.7)	823 (21.7)	<0.001
**Co-medication**						
Anticholinergic agents	554 (8.6)	614 (9.5)	0.032	333 (8.8)	389 (10.3)	0.05
Hypnotics	1,517 (23.5)	1754 (27.2)	0.084	1,057 (27.9)	1,062 (28)	0.003
Corticosteroids	277 (4.3)	307 (4.8)	0.022	152 (4)	165 (4.4)	0.017
H2-receptor antagonists	1,061 (16.4)	1,382 (21.4)	0.127	714 (18.8)	729 (19.2)	0.01
Silymarin	3,567 (55.2)	3,389 (52.5)	0.055	2064 (54.4)	1989 (52.5)	0.04
ACEI/ARB	1742 (27)	1905 (29.5)	0.056	1,125 (29.7)	1,123 (29.6)	0.001
Beta-blockers	1,139 (17.6)	1,419 (22)	0.109	712 (18.8)	723 (19.1)	0.007
CCB	1907 (29.5)	1870 (28.9)	0.013	1,187 (31.3)	1,186 (31.3)	0.001
Diuretics	462 (7.2)	606 (9.4)	0.081	285 (7.5)	330 (8.7)	0.043
Antiplatelet agents	806 (12.5)	808 (12.5)	0.001	478 (12.6)	493 (13)	0.012
Anticoagulants	66 (1)	123 (1.9)	0.074	44 (1.2)	38 (1)	0.015
Statins	587 (9.1)	665 (10.3)	0.041	356 (9.4)	384 (10.1)	0.025
Fibrates	106 (1.6)	96 (1.5)	0.012	60 (1.6)	68 (1.8)	0.016
**Insured premium**						
<22800 TWD	3,615 (56)	3,291 (50.9)	0.101	2199 (58)	2090 (55.1)	0.058
22800–36299 TWD	1,490 (23.1)	1,647 (25.5)	0.057	768 (20.3)	926 (24.4)	0.1
≥36300 TWD	1,356 (21)	1,523 (23.6)	0.062	825 (21.8)	776 (20.5)	0.032
**Urbanization level**						
Urban	1,203 (18.6)	1,285 (19.9)	0.032	676 (17.8)	687 (18.1)	0.008
Suburban	2028 (31.4)	2074 (32.1)	0.015	1,170 (30.9)	1,140 (30.1)	0.017
Rural	3,230 (50)	3,102 (48)	0.04	1946 (51.3)	1965 (51.8)	0.01
**Treatment duration**						
Week, mean ± SD	22.94 ± 1.29	13.66 ± 4.22	2.974	22.93 ± 1.34	14.6 ± 4.98	2.28
**IFN therapy (±RBV)**						
PEGIFN α-2a	4,189 (64.8)			4,246 (65)		
Others^*^	2272 (35.2)			2285 (35)		
**DAA therapy (±RBV)**						
DCV + ASV		807 (12.5)			721 (19)	
OBV + PTV/r + DSV		2136 (33.1)			1971 (52.1)	
EBR + GZR		779 (12.1)			273 (7.2)	
SOF + LDV		1,128 (17.5)			408 (10.8)	
SOF		1,611 (24.9)			413 (10.9)	

IFN, interferon; DAA, direct-acting antiviral agents; SMD, standardized mean difference; HBV, hepatitis B virus; HIV, human immunodeficiency virus; H2-receptor antagonists, histamine 2 receptor antagonists; ACEI, angiotensin-converting enzyme inhibitors; ARB, angiotensin receptor blockers; CCB, calcium channel blockers; PEGIFN, peginterferon; DCV, daclatasvir; ASV, asunaprevir; OBV, ombitasvir; PTV/r, paritaprevir/ritonavir; DSV, dasabuvir; EBR, elbasvir; GZR, grazoprevir; SOF, sofosbuvir; LDV, ledipasvir.

* : PEGIFN α-2b or IFN α-2a.

### 3.2 Impact of DAAs on the risk of neuropsychological disorders

**FIGURE 3:**
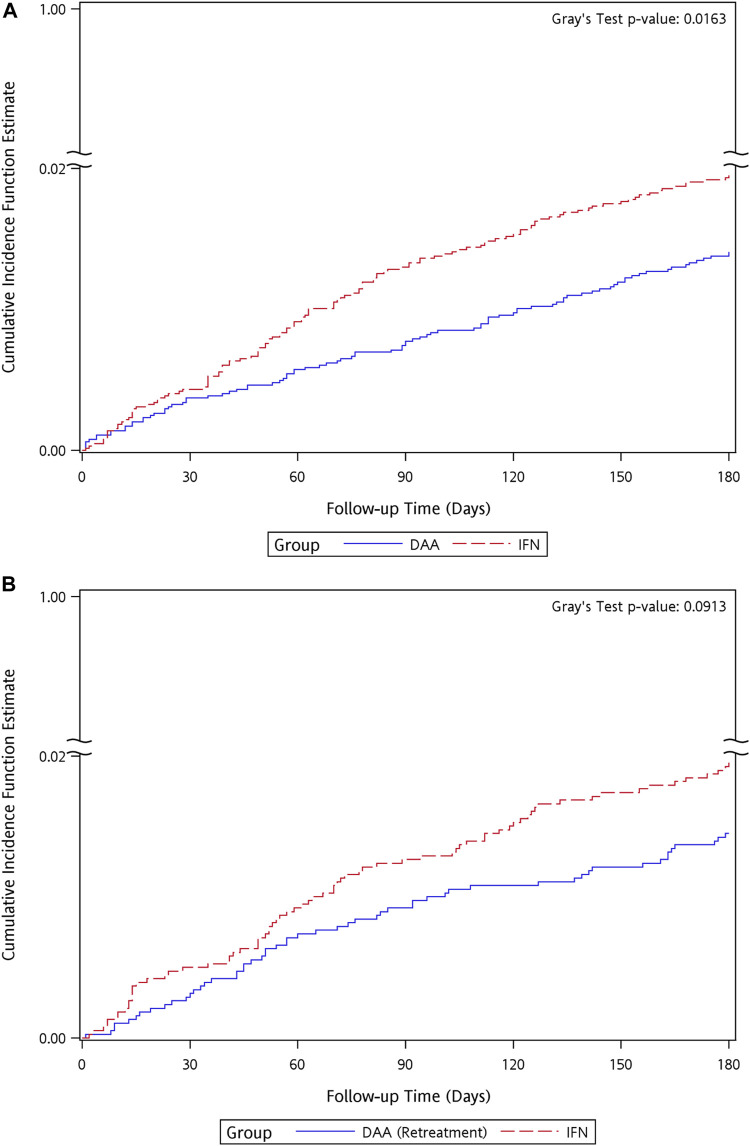
Kaplan–Meier curves of cumulative event rate of neuropsychological disorders for the matched cohort of IFN group and **(A)** Tx-naïve DAA, **(B)** re-Tx DAA group.

Among the cohorts of Tx-naïve DAA group and re-Tx DAA group and their matched IFN controls, patients who developed NPDs were predominantly censored at the time of the occurrence of mood and anxiety disorders. There were 91 (1.41%) patients in the Tx-naïve DAA group and 126 (1.95%) patients in the IFN group with incidences of NPDs during the follow-up period, with crude incidence rates (IR) of 2.38 and 3.3 per 1,000 person-months, respectively. There was a difference observed in the cumulative incidence curves between the Tx-naïve DAA group and IFN group (Gray’s test *p* = 0.0163) (Figure 3). In the cohort of the re-Tx DAA group and their matched IFN controls, 55 (1.45%, IR = 2.45 per 1,000 person-months) patients in the re-Tx DAA group and 74 (1.95%, IR = 3.31 per 1,000 person-months) patients in the IFN group developed incidences of NPDs. No difference was found in the cumulative incidence curves for the re-Tx DAA group and IFN group (Gray’s test *p* = 0.0913) (Figure 3).

Patients in the DAA group had a lower risk than the matched controls in the IFN group (crude hazard ratio [HR] = 0.72, 95% CI = 0.55–0.94, *p* = 0.017), and the results remained significant after adjusting for gender, age group, and usage of anticholinergic agents and hypnotics (adjusted HR [aHR] = 0.69, 95% CI = 0.52–0.9, *p* = 0.006) (Table 2.). Among the components of the primary outcome, a lower risk of mood and anxiety disorders (HR = 0.68, 95% CI: 0.52–0.91, *p* = 0.009) was found in the Tx-naïve DAA group as than in the IFN group. There was no difference in the risk of other NPDs between the Tx-naïve DAA group and IFN group (HR = 1.11, 95% CI = 0.45–2.73, *p* = 0.819). No significantly lower risk of developing NPDs and their components for secondary outcome was observed in the re-Tx DAA group than in the IFN group.

**TABLE 2:** Incidence and hazard ratio of neuropsychological disorders.

	IFN group			DAA group			Univariate analysis	*p*-value	Multivariate analysis	*p*-value
	Event (%)	PM	IR	Event (%)	PM	IR	Crude HR (95% CI)		Adjusted HR^a^ (95% CI)	
**Cohort I**	**Tx-naïve group (n = 6,461)**			**Tx-naïve group (n = 6,461)**						
Overall (any NPDs)	126 (1.95)	38142.8	3.3	91 (1.14)	38271.9	2.38	0.72^*^ (0.55–0.94)	0.017	0.69^**^ (0.52–0.9) ^∆^	0.006
Mood and anxiety disorders	118 (1.83)	38348.6	3.08	81 (1.25)	38498.9	2.1	0.68^**^ (0.52–0.91)	0.009	0.65^**^ (0.49–0.86)	0.003
Other NPDs	9 (0.14)	38728.9	0.23	10 (0.15)	38740	0.26	1.11 (0.45–2.73)	0.819	1.06 (0.43–2.62)	0.897
**Cohort II**	**Tx-naïve group (n = 3,792)**			**re-Tx group (n = 3,792)**						
Overall (any NPDs)	74 (1.95)	22357.5	3.31	55 (1.45)	22445.8	2.45	0.74 (0.52–1.05)	0.092	0.74 (0.52–1.05)^▲^	0.091
Mood and anxiety disorders	70 (1.85)	22505.9	3.11	50 (1.32)	22584.6	2.21	0.71 (0.5–1.02)	0.067	0.72 (0.5–1.04)	0.077
Other NPDs	4 (0.11)	22736	0.18	5 (0.13)	22731.1	0.22	1.25 (0.34–4.66)	0.739	1.25 (0.34–4.66)	0.737

PM, person-month; IR, incidence rate (number of event/per 1,000 person-month); HR, hazard ratio;^*^: *p* < 0.05;^**^: *p* < 0.01; ^***^: *p* < 0.001.

a : Adjusted covariates: age group, gender, and baseline variables with significance in univariate analysis which finally enter the regression model after stepwise selection.

∆: Adjusted covariates: age, gender, and co-medication (anticholinergic agents and hypnotics); ^▲^: Adjusted covariates: age, gender, and co-medication (hypnotics).

Tx-naïve group, treatment-naïve CHC patients; re-Tx group, retreatment patients who received DAA and had a prior IFN failure.

### 3.3 Factors associated with the risk of developing neuropsychological disorders

Cox regression analysis for risk factors of NPDs is presented in Table 3. In univariate analysis, among the matched cohort of the Tx-naïve DAA group and IFN group, male patients had a significantly lower risk of developing NPDs than female patients (HR = 0.68, 95% CI: 0.52–0.89, *p* = 0.005). Hypertension and medication use of anticholinergic agents, hypnotics, and histamine 2 receptor antagonists were significantly associated with an increased risk. Only factors of male gender and usage of anticholinergic agents and hypnotics were selected in stepwise multiple regression analysis and included in the multivariate model. Similar results were observed among the matched cohort of the re-Tx DAA group and IFN group, and male gender was also significantly associated with a lower risk of NPDs (HR = 0.6, 95% CI: 0.44–0.82, and *p* = 0.001).

**TABLE 3:** Cox cause-specific hazards model for risk factors of neuropsychological disorders among two cohorts.

	Cohort I					Cohort II			
	Crude HR (95% CI)	*p*-value	Adjusted HR^∆^ (95% CI)	*p*-value	Crude HR (95% CI)		*p*-value	Adjusted HR^∆^ (95% CI)	*p*-value
**Gender**									
Female	1 (reference)		1 (reference)		1 (reference)			1 (reference)	
Male	0.68^**^ (0.52–0.89)	0.005	0.72^*^ (0.55–0.95)	0.019	0.56** (0.39–0.8)		0.002	0.6^**^ (0.41–0.86)	0.006
**Age group**									
Age <65	1 (reference)		1 (reference)		1 (reference)			1 (reference)	
Age >65	1.14 (0.87–1.5)	0.345	1.07 (0.81–1.41)	0.652	1.08 (0.76–1.53)		0.678	1.03 (0.72–1.46)	0.889
**Comorbidity**									
Hypertension	1.41 (1.02–1.94)	0.037			1.66 (1.12–2.45)		0.012		
Diabetes mellitus	1.48 (1–2.19)	0.049			1.54 (0.96–2.45)		0.072		
Autoimmune diseases	1.79 (1.02–3.14)	0.041			0.91 (0.34–2.47)		0.857		
**Co-medication**									
Anticholinergic agents	2.53^***^ (1.81–3.53)	<0.001	2.06^***^ (1.47–2.9)	<0.001	1.36 (0.81–2.29)		0.252		
Hypnotics	2.74^***^ (2.1–3.58)	<0.001	2.46^***^ (1.87–3.23)	<0.001	2.34*** (1.65–3.3)		<0.001	2.23^***^ (1.58–3.16)	<0.001
H2-receptor antagonists	1.43 (1.05–1.95)	0.0224			1.47 (0.99–2.19)		0.055		
Silymarin	1.04 (0.8–1.36)	0.7599			1.47* (0.99–2.19)		0.013		
ACEI/ARB	1.32 (1–1.75)	0.0524			1.72** (1.21–2.44)		0.002		
Antiplatelet agents	1.45 (1.02–2.07)	0.039			1.58 (1.01–2.45)		0.045		

HR, hazard ratio; *: *p* < 0.05; **: *p* < 0.01; ***: *p* < 0.001.

∆: Adjusted for age group, gender, and baseline variables with significance in univariate analysis which finally enter the regression model after stepwise selection.

**FIGURE 4:**
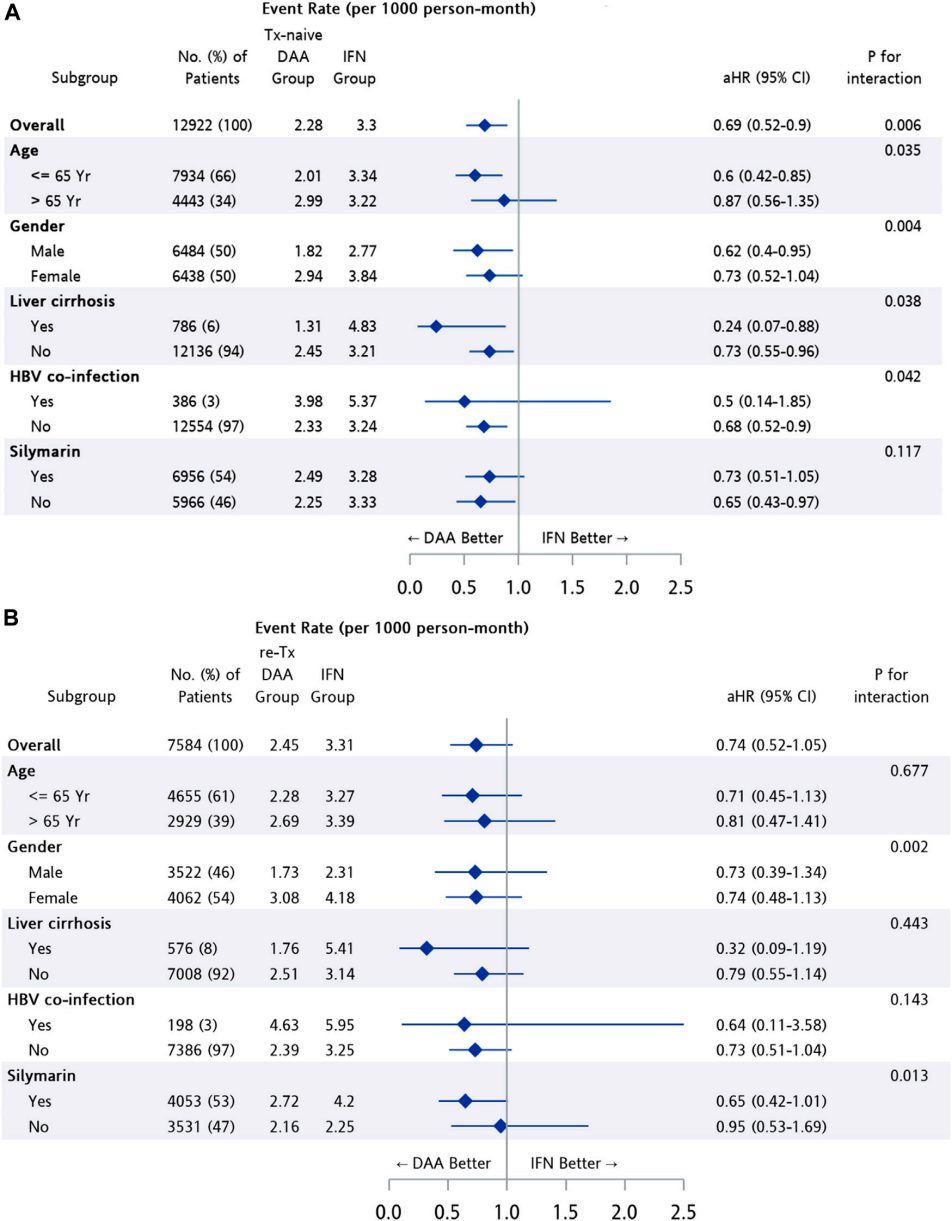
Subgroup analysis among patients from matched cohort of IFN group and **(A)** Tx-naïve DAA, **(B)** re-Tx DAA group.

The difference in risk of NPDs between treatment groups was further analyzed after stratification (Figure 4). Patients in the Tx-naïve DAA group had a significantly lower risk than those in the IFN group among subpopulations of patients with age ≤65 years (95% CI: 0.42–0.85, *p* = 0.005), male patients (95% CI: 0.4–0.95, *p* = 0.028), and patients with cirrhosis (95% CI: 0.07–0.88, *p* = 0.038). The risk of developing NPDs was found to be similar for the re-Tx DAA group and the IFN group, regardless of age, gender, and status of cirrhosis.

## 4 Discussion

In the current nationwide cohort study, more than 96% patients in both the Tx-naïve and re-Tx DAA groups completed planned treatment duration; and nearly all controls (99.8%) in the IFN group for the Tx-naïve and re-Tx DAA groups had an adherence rate of more than 80%. Therefore, the included CHC patients were suggested to have good adherence to HCV treatment. According to the official report from the Taiwan National Hepatitis C Program Office, a high SVR rate of more than 97% was achieved among patients who were reimbursed with DAA treatment with adequate follow-up in 2017 and 2018 (National Hepatitis C Program Office et al, 2020). In the present study, only patients with more than 6-week supply from their DAA prescriptions were included in the DAA group, which indicated that the patients with poor virological response at week 4 were excluded. Theoretically, both the Tx-naïve and re-Tx DAA groups had a high SVR rate similar to the reported rate provided by the National Hepatitis C Program Office. Among patients receiving IFN therapy, the positive predictive value (PPV) of RVR on SVR was more than 86% across all genotypes (Yu et al., 2007; Martinot-Peignoux et al., 2009; Fried et al., 2011). Under the policy of Taiwan’s NHI system, the IFN treatment is reimbursed for up to 24 weeks for patients with RVR and not more than 16 weeks for those who fail to achieve EVR. Therefore, only patients with cumulative supply of IFN prescriptions between 17 and 24 weeks during a period of 17–28 weeks could be included in the IFN group. It is suggested that the patients included in the IFN group were representative of IFN-treated CHC patients with RVR, and approximately 86% patients of them achieved SVR based on the PPV of RVR. However, in comparison to the SVR rate of approximately 97% among the patients in the DAA group, more patients failed HCV treatment in the IFN group.

The distribution of HCV genotypes might be different between the study groups. The predominant HCV genotypes in Taiwan were type 1b and type 2 (Polaris Observatory HCV Collaborators, 2017). The HCV genotype is an important predictor of the treatment response for patients receiving IFN therapy; lower RVR rates were observed among those infected with genotype 1 than with other genotypes. Accordingly, there was a high probability that more patients with HCV genotype 2 infection were included in the IFN group. In contrast, the predominant HCV genotypes in both DAA groups tended to be genotype 1 based on their reimbursed DAA regimens.

In the previous study including CHC patients treated with IFN, the incidence of major psychiatric disorders and affective disorders did not differ between the SVR group and non-SVR group (Tsai et al, 2020), whereas the present study showed a lower risk in both of the overall NPDs (HR = 0.72, 95% CI: 0.55–0.94, *p* = 0.017) and the components of the primary outcome, the mood and anxiety disorders (HR = 0.68, 95% CI: 0.52–0.91, *p* = 0.009), among treatment-naïve CHC patients receiving DAA treatment. These results suggested that successful antiviral therapy with the DAA regimen was associated with the benefits in reducing the risk of the NPDs, particularly for the component of mood and anxiety disorders, as compared with IFN therapy among treatment-naïve CHC patients in the 6-month period after treatment completion. The result might be explained by the fact that neuropsychological disturbances were reduced by the high efficacy of DAA treatment on HCV eradication. There might be concerns that the lower risk observed in DAA-treated patients in the present study was relative to adverse neuropsychiatric effects of IFN therapy. Psychiatric symptoms including depression and anxiety are commonly reported in CHC patients treated with IFN, which is a major reason for discontinuing IFN therapy (Zdilar et al., 2000; Dieperink et al., 2003; Horikawa et al., 2003; Kraus et al., 2003; Gleason et al., 2007). In contrast, high tolerability was demonstrated in DAA treatment (Götte and Feld, 2016). However, a meta-analysis showed the majority of new incidences of depressive symptoms as well as anxiety symptoms occurred between 4 and 12 weeks of IFN treatment and few occurred after 24 weeks of treatment (Udina et al., 2012). The incidences in the present study were predominantly anxiety disorders, and patients who experienced study outcomes during treatment were excluded. Therefore, the incidences identified in the follow-up period after the end of the treatment were less likely to be associated with the adverse effects of IFN treatment.

There was no significant difference between the Tx-naïve DAA group and IFN group in the risk of other NPDs which included psychotic disorders and cognitive disorders (HR = 1.11, 95% CI: 0.45–2.73, *p* = 0.819). However, improving cognitive and neuropsychological outcomes among the CHC patients achieving SVR has been shown in several studies conducted using self-control scale measurement in a short follow-up time (Barbosa et al., 2017; Evon et al., 2019; Hassaan et al., 2019; Kesen et al., 2019; Fabrazzo et al., 2020; Vaghi et al., 2020; Ibáñez-Samaniego et al, 2022; Kaur et al., 2022; Mahran et al., 2022). The previous studies evaluated differences in mean scores of symptom clusters between pre-treatment and post-treatment assessment, at 12 weeks to 6 months after therapy completion. In contrast to the present study evaluating the risk reduction in NPDs, these studies suggested HCV elimination was associated with an improvement in severity of anxiety, depression, and cognitive disturbance. Due to the neuropsychological outcomes being evaluated with the presence of diagnosis records in the present study, the 6-month follow-up period might not be adequate to observe the occurrence of dementia and psychosis and reveal meaningful patterns. Therefore, the influence of DAA on the incidence of psychotic disorders and cognitive disorders among CHC patients remained inconclusive.

In contrast to the Tx-naïve DAA group, the re-Tx DAA group failed to achieve a lower risk for NPDs. This observation might be explained by the persistence of HCV infection, which was theoretically more long-lasting in the retreatment group as than in the Tx-naïve group. Since the HCV has been hypothesized to induce neuropsychological manifestations by a direct neurotoxic effect of CNS infection, or by secondary effects of the chronic inflammation, a longer persistence of HCV infection was associated with an increased risk of developing neuropsychological dysfunctions among CHC patients in theory. Another plausible explanation was that the reason for IFN failure among patients in the re-Tx DAA group might be the treatment discontinuation due to neuropsychiatric problems. Therefore, the failure to achieve significant risk reduction on NTDs with DAA in the re-Tx group might result from the pre-existing neuropsychological condition without seeking medical care before initiation of DAA therapy.

A gender-specific difference in the risk of NPDs was observed in the study cohorts. Male gender was associated with a reduced risk of NPDs when compared with female gender in both the univariate and multivariate analyses. A similar result was observed in the previous study evaluating the risk of major psychosis among CHC patients, which suggested that female gender was the risk factor for affective psychosis (aHR = 4.27, 95% CI = 1.31–13.89, *p* = 0.016). (Tsai et al, 2020).

In subgroup analysis, the result suggested that the better neuropsychological outcome after DAA treatment in comparison with IFN treatment was more pronounced among treatment-naïve CHC patients with age ≤65 years (aHR = 0.6, 95% CI: 0.42–0.85, *p* = 0.005), male gender (aHR = 0.62, 95% CI: 0.4–0.95, *p* = 0.028), and liver cirrhosis (aHR = 0.24, 95% CI: 0.07–0.88, *p* = 0.038). Additionally, lower risk of the NPDs was observed among treatment-naïve CHC patients in both cirrhotic and non-cirrhotic subpopulations. The result corresponded to the finding in a study investigating depression, anxiety, and quality of life among CHC patients with the self-control scale, which showed improvement on anxiety score among patients after completion of DAA treatment in both cirrhotic and non-cirrhotic groups. (Kesen et al, 2019).

There were some limitations in the present study that need to be addressed. First, treatment success in patients could not be confirmed since the information on SVR status was not available in the database. Second, there was lack of information on HCV genotypes and baseline viral titers for each patient. Third, racial disparities in response to antiviral therapy could not be examined. A previous study showed the difference in DAA treatment outcomes for individuals originating from countries across Africa (Aranday-Cortes et al, 2022). Therefore, the influence of SVR status, infecting genotype viral load, and race on the study outcomes was not evaluated.

In conclusion, DAA therapy is suggested to reduce the risk of developing NPDs as compared with IFN therapy among treatment-naïve CHC patients in a 6-month period after treatment completion, especially among patients with age ≤65 years), male gender, or cirrhosis. However, no significant difference was revealed between DAA-treated patients with prior IFN failure and IFN-treated patients pertaining to neuropsychological disorders. Data reporting over longer follow-up periods will be required (Lim et al., 2012).

## Data Availability

The data analyzed in this study are subject to the following licenses/restrictions: the corresponding author had full access to all the data in the study and takes responsibility for the integrity of the data and the accuracy of the data analysis. Data are available from the National Health Insurance Research Database (NHIRD) published by the Bureau of National Health Insurance (BNHI) of the Ministry of Health and Welfare. Owing to the legal restrictions imposed by the Government of Taiwan related to the Personal Information Protection Act, the database cannot be made publicly available. The conclusions presented in this study are those of the authors and do not necessarily reflect the views of the BNHI, the Ministry of Health and Welfare. Requests to access these datasets should be directed to P-CL, pclin@vghks.gov.tw.
