# Supplementary material for: Neuropsychiatric disorders in chronic hepatitis C patients after receiving interferon or direct-acting antivirals: a nationwide cohort study
Source: Front Pharmacol. 2023 Jul 19;14:1191843. doi: 10.3389/fphar.2023.1191843 (PMC10394649; doi:10.3389/fphar.2023.1191843)
Supplement: Supplementary file 1 [file DataSheet1.docx]

# Appendices

1. Diagnosis codes for comorbidities

| **Comorbidities** | **ICD-9 CM/ ICD-10 CM** |
| --- | --- |
| Hypertension | 401-405/ I10-I15 |
| Hyperlipidemia | 272.0-272.5/ E78.0-E78.5 |
| Diabetes mellitus | 250/ E10-E14 |
| Chronic kidney disease | 585/ N18 |
| Ischemic heart disease | 410-414/ I20-I25 |
| Cerebrovascular disease | 430-438/ G45-G46, I60-I69 |
| Cirrhosis | 571.2, 571.5-571.6/ K70.2-K70.3, K74 |
| Liver cancer | 155/ C22 |
| HBV coinfection | 070.2-070.3/ B16, B18.0-B18.1, B19.1, V02.61, Z22.51 |
| HIV coinfection | 042-044/ B20-B22, B24 |
| Autoimmune disorder | 135, 273, 242.0, 245.2, 255.4, 287.3, 279.4, 279.8, 283.0, 340, 358.0, 443.0, 555.9, 558.9, 571.49, 576.1, 579.0, 696.0, 697.0, 710.0, 710.1, 710.3, 710.9, 720.0, 996.8/ D86, D89, D59.0-D59.1, D69.3, E05.0, E06.3, E27.1, G35, G70.0, I73.0, K50.9, K52.9, K74.3, K74.5, K83.0, K90.0, L40.0-L40.2, L40.5, L40.8, L43, M05, M06, M08.1, M32, M33.1-M33.2, M33.9, M34, M35.0, M35.9, M45 |

1. The medications identified as anticholinergic agents, hypnotics, and corticosteroids

| **Co-medication** | **medications (ATC code)** |
| --- | --- |
| Anticholinergic agents | Disopyramide, Amitriptyline, Amoxapine, Clomipramine, Desipramine, Doxepin, Imipramine, Nortriptyline, Paroxetine, Protriptyline, Trimipramine, Darifenacin, Fesoterodine, Flavoxate, Oxybutynin, Solifenacin, Tolterodine, Trospium, Prochlorperazine, Promethazine, Benztropine, Trihexyphenidyl, Brompheniramine, Carbinoxamine, Chlorpheniramine, Clemastine, Cyproheptadine, Dexbrompheniramine, Doxylamine, Dexchlorpheniramine, Dimenhydrinate, Diphenhydramine, Hydroxyzine, Meclizine, Clidinium-chlordiazepoxide, Dicyclomine, Homatropine, Hyoscyamine, Methscopolamine, Propantheline, Chlorpromazine, Clozapine, Loxapine, Olanzapine, Perphenazine, Thioridazine, Trifluoperazine, Cyclobenzaprine, and Orphenadrine |
| Hypnotics | Benzodiazepine derivatives (N05BA), Benzodiazepine derivatives (N05CD), Benzodiazepine related drugs (N05CF) |
| Corticosteroids | Systemic use: Mineralocorticoids (H02AA),Glucocorticoids (H02AB), combinations (H02B) |
|  | |

1. Diagnosis codes for study outcome

|  | **ICD-9 CM** | **ICD-10 CM** |
| --- | --- | --- |
| **Mood and anxiety disorders** |  |  |
| Depressive disorders | 296.2–296.3, 300.4, 311 | F32, F33, F34.1 |
| Mania | 296.0, 296.1, 296.81 | F30 |
| Bipolar disorders | 296.4–296.6 | F31 |
| Anxiety disorders | 300.0, 300.2 | F40, F41 |
| **Psychotic disorders** |  |  |
| Schizophrenia, schizotypal and delusional disorders | 295, 297, 298 | F20–F29 |
| **Cognitive disorders** |  |  |
| Alzheimer's disease | 331.0 | F00, G30 |
| Dementia | 290.0–290.4, 290.8, 290.9, 294.1 | F01–F03 |
| Delirium | 293.0, 293.1 | F05 |
